# Observation of Boson Peak of Fragile Baltic Amber Glass by Terahertz Time-Domain Spectroscopy

**DOI:** 10.3390/ma17235956

**Published:** 2024-12-05

**Authors:** Toru Sasaki, Soo Han Oh, Tatsuya Mori, Seiji Kojima

**Affiliations:** Department of Materials Science, University of Tsukuba, Tsukuba 305-8573, Ibaraki, Japan; torusasaki41@gmail.com (T.S.); s2030075@s.tsukuba.ac.jp (S.H.O.); mori@ims.tsukuba.ac.jp (T.M.)

**Keywords:** glass, fragile, boson peak, Baltic amber, terahertz time-domain spectroscopy

## Abstract

Amber is a fragile (in Angell’s classification) natural glass that has performed maturation processes over geological time. The terahertz dynamics of Baltic amber that was about 40 million years old were studied by terahertz time-domain spectroscopy (THz-TDS) in the frequency range of 0.2 and 6.0 THz. In general, the intensity of a boson peak is weak for fragile glass. In the terahertz transmission spectra of Baltic amber in the previous study, no boson peak was observed upon slow cooling. However, in the present study, upon rapid cooling down to 15 K, the boson peak was observed clearly at 0.36 THz by the suppression of ice nucleation of confined water. The dynamic correlation length determined by the boson peak frequency was compared with the static structure correlation length and the scale of the medium-range order as determined by the first sharp diffraction peak of X-ray diffraction (XRD) in the recent literature. It was found that the dynamic correlation length determined by THz-TDS was closely related to the static correlation length determined by the XRD analysis.

## 1. Introduction

The boson peak (BP) is a universal feature of vibrational dynamics in glasses in the terahertz (THz) frequency range [1,2,3]; it has been observed by Raman scattering, far-infrared spectroscopy, inelastic neutron/X-ray scattering spectroscopy, and THz time-domain spectroscopy (THz-TDS). A BP is ascribed to the excess vibrational density of states (VDoS) g(*E*), which produces a peak in the g(*E*)/*E*^2^ deviated from the Debye model. The excess heat capacity was also observed as a bump in *C*_p_/*T*^3^ at low temperatures, where *C*_p_ is a heat capacity. Sokolov et al. reported the correlation between a BP frequency and the first sharp diffraction peak (FSDP) in a static structure factor S(Q) of diffraction measurements, where Q is a wave vector [4]. There are two typical contributions to the low-frequency excitation spectra (Raman and neutron scattering, specific heat, etc.) of glass formers: relaxations and vibrations (BPs). It is shown from the analysis of low-frequency Raman spectra that the relative weight of vibrational over relaxational excitations is larger for less fragile (in Angell’s classification) glass formers. Therefore, as the fragility increases, the intensity of the relaxation process increases, while the BP intensity decreases. For a strong glass such as B_2_O_3_, the BP intensity is intense, whereas for a fragile glass such as Ca_2_Ka(NO_3_)_7_ (CKN), the BP intensity is very weak [5]. Amber is a typical fragile glass with a fragility index of 90 [6]. In Baltic amber, a BP was not observed by the standard experiment by THz-TDS [7]. However, the BP of the 110-million-year-old amber from El Soplao (Spain) was investigated by the specific heat, *C*_p_, at low temperatures, and the peak of *C*_p_/*T*^3^ was observed at about 3 K [8]. Recently, the BP of a Spanish amber was observed at 1.5 meV by an inelastic X-ray scattering experiment [6].

Glasses are nonequilibrium states whose microscopic properties vary depending on their thermal history. The amber is formed via a fossilization process by cross-linking of the original organic resin via free radical polymerization [8,9]. Amber is aged for many million years at temperatures well below a glass transition temperature *T*_g_ [10,11]. Amber is a unique example of a glass that has been aging for a very long time below its *T*_g_, thus reaching a thermodynamically stable state that is not accessible under normal experimental conditions [12]. The *T*_g_ of amber does not show a large variation. The nine pieces of amber with different ages from different locations were investigated by differential scanning calorimetry, and the *T*_g_ was determined to be between about 390 K and 460 K [12].

Anderson et al. reported a classification of fossil resins and Baltic amber belonging to the Class I category [10]. The bulk of Baltic amber consists of a polymer of the labdatrienoid compounds, including communic acid, and it is communal with its hydroxyl groups being partially succinylated. The amber also contains a few percent of many other compounds such as terpinol, succinate, and hemisuccinate esters [13]. The density of Baltic amber is 1.0~1.1 g/cm^3^, and its refractive index is 1.54. The melting temperature is 250~300 °C, and the glass transition temperature is 182 °C [12]. The BP and medium-range order (MRO) of structural disorder in Baltic amber glass has not yet been studied.

Recently, the generation of a coherent THz-wave radiation has become possible by recent progress in a femtosecond pulse laser. The combination of the compact photoconductive antennas driven by femtosecond laser pulses enables THz-TDS [14,15]. The measurement of the amplitude and phase of coherent transmitted THz waves enables the accurate determination of the real and imaginary parts of a dielectric susceptibility in a THz range. THz-TDS has been applied to various kinds of materials such as ferroelectrics [16], semiconductors [17], superconductors [18], drug materials [19,20], and glassy materials [20,21]. In the present study, the temperature dependence of vibrational properties of Baltic amber glass was studied by the terahertz time-domain spectroscopy in the THz range between 0.2 and 6.0 THz.

## 2. Materials and Methods

**Figure 1:**
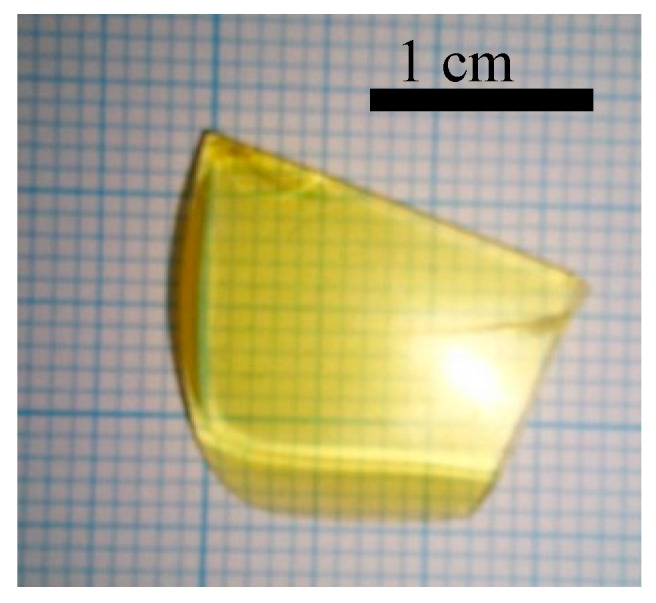
Baltic amber.

The archaeological ambers (Figure 1) we measured were obtained in the Baltic Sea, and the age of the ambers is about 40 million years old. The ambers were cut and polished for THz-TDS measurements, and the thicknesses of the samples were 2.311 and 0.912 mm ± 0.0005 mm to obtain the appropriate intensity of THz transmission spectra.

**Figure 2:**
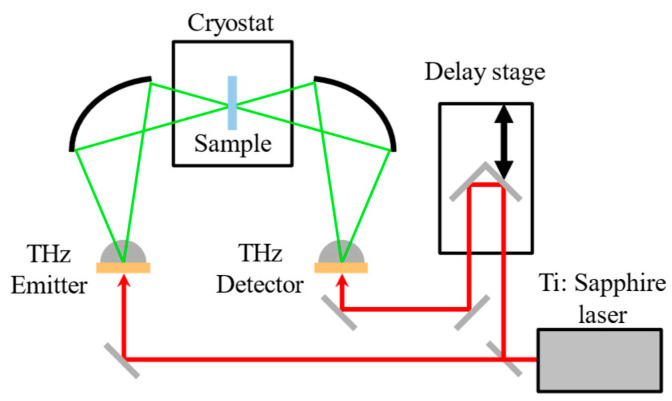
Experimental setup of the THz time-domain spectrometer (RT-10000).

A terahertz time-domain spectroscopy (THz-TDS) system (RT-10000, Tochigi Nikon Co., Otawara, Japan) and liquid-helium flow cryostat system (Helitran LT-3B, Advanced Research Systems, Pelkie, MI, USA) [3,15] were used with the standard transmission configuration for temperature-dependent measurements in the frequency range of 0.2~4.0 THz, and the temperature ranged from 13 to 295 K, as shown in Figure 2. Low-temperature grown GaAs photoconductive (PC) antennas were utilized for the THz pulse emitter and detector, and the PC antennas were triggered by a mode-locked Ti: a sapphire pulsed laser with a wavelength of 780 nm, a pulse width of less than 100 fs, and a repetition rate of 80 MHz. The THz-wave propagation path was enclosed in a dry air chamber within which dry air flowed. The broadband spectra between 0.2 and 6.5 THz at room temperature were measured by another THz-TDS system (TAS7500SU, Advantest Corp., Tokyo, Japan) in the frequency range of 0.5~6.5 THz. In the data analysis at room temperature, we smoothly connected the absolute value of the complex dielectric constants of the same sample at 1.9 THz, based on the low-frequency data.

**Figure 3:**
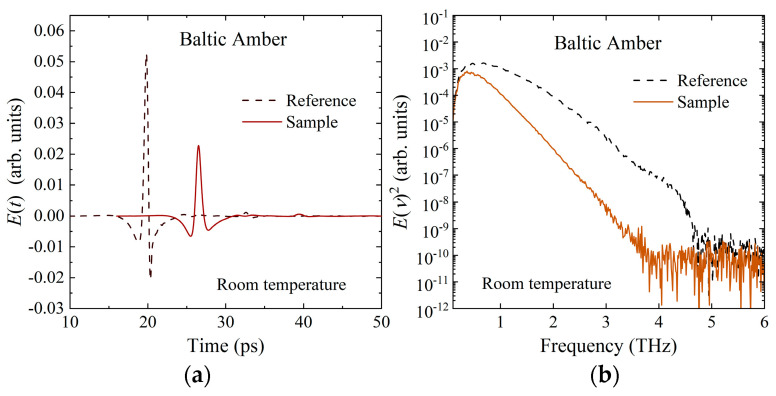
Time-domain and frequency-domain responses of (**a**) *E*(t) and (**b**) *E*(ν)^2^ of Baltic amber at room temperature.

The measured time-domain THz E-field waveforms transmitted through the air (reference) and the amber sample with a thickness of 2.311 mm are shown in Figure 3a. The frequency–domain power spectra of a reference and the sample are shown in Figure 3b.

The THz transmission $\tilde{t}\left(\nu \right)$ was calculated by the measured time-domain waveforms shown in Figure 3a,b using the following equation,

(1) $$\tilde{t}\left(\nu \right)=\frac{{\tilde{E}}_{sam}\left(\nu \right)}{{\tilde{E}}_{ref}(\nu )}=\frac{4\tilde{n}}{{\left(\tilde{n}+1\right)}^{2}}\exp\left\{i\frac{2\pi \nu }{c}\left(\tilde{n}-1\right)d\right\},$$

where *E*_sam_(ν) and *E*_ref_(ν) are the amplitude spectra of the THz pulse transmitted by the sample and the reference, respectively. $\tilde{n}$, c, and *d* are the complex refractive index, the light velocity, and the thickness of a sample, respectively. The complex dielectric constant ε(ν) = ε′(ν) + iε″(ν) is determined by

(2) $${\tilde{n}}^{2}\left(\nu \right)={\left(n\left(\nu \right)+i\kappa \left(\nu \right)\right)}^{2}=\tilde{\varepsilon }\left(\nu \right)=\varepsilon^{\prime }\left(\nu \right)+i\varepsilon^{\prime \prime }\left(\nu \right).$$

This frequency range is dominated by the universal feature of glasses, the so-called BP arising from an excess in the vibrational density of state (VDoS) [1,2]. The imaginary parts of the complex dielectric constant are related to the VDoS g(ν) through the infrared (IR) coupling constant *C*_IR_(ν), and a BP spectrum is expressed by the following equation [3]

(3) $$\frac{2\pi \cdot \varepsilon \prime \prime (\nu )}{c\cdot n(\nu )\cdot \nu }=\frac{\alpha \left(\nu \right)}{\nu^{2}}=C_{IR}\left(\nu \right)\cdot \frac{g\left(\nu \right)}{\nu^{2}},$$

where *C*_IR_(ν) is the IR coupling constant. In various kinds of glasses, BPs were observed as broad asymmetric peaks in the sub-THz spectra of α(ν)/ν^2^ or ε″(ν)/(*n*(ν)·ν) by THz-TDS [3,18].

## 3. Observation of Boson Peak in the Sub-THz Range

**Figure 4:**
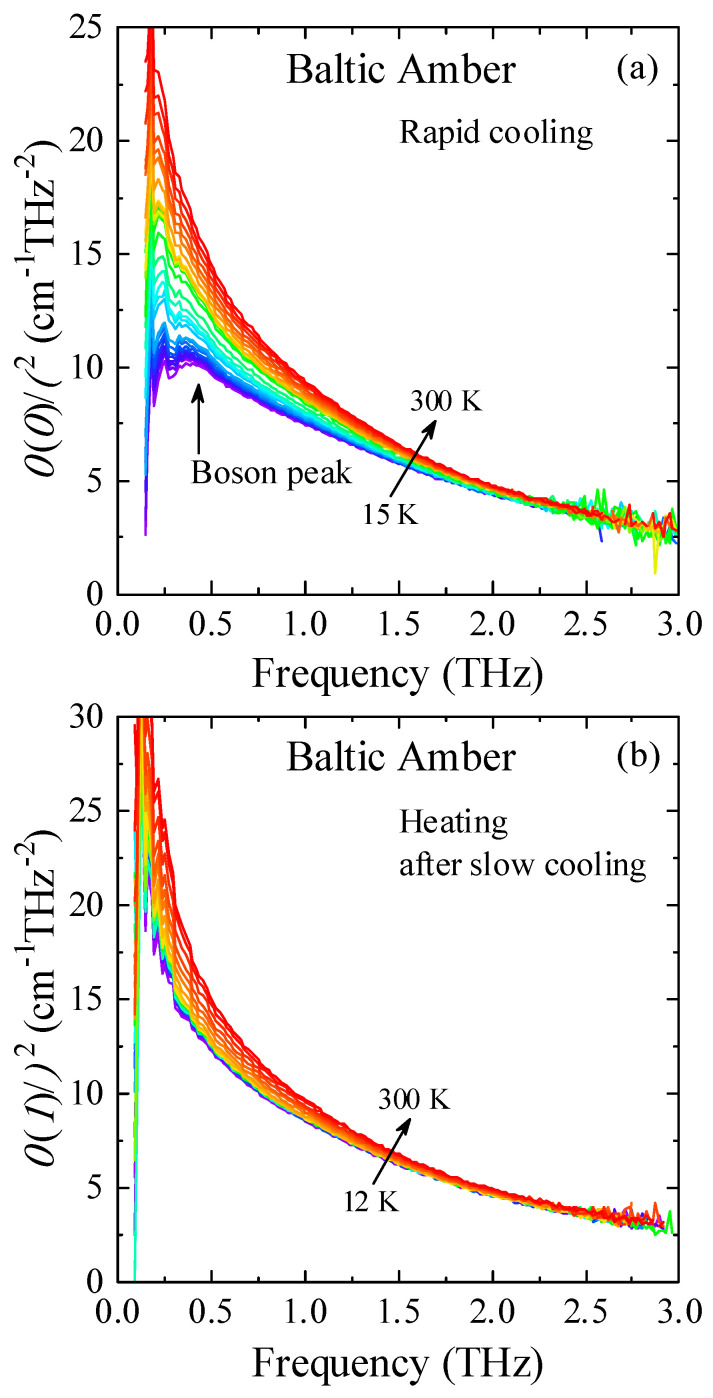
(**a**) Temperature dependence of THz-TDS spectra α(ν)/ν^2^ of Baltic amber observed after a rapid quench of 5 K/min down to 15 K. Upon heating after the quench, the intensity of a BP gradually decreases. (**b**) Temperature dependence of THz-TDS spectra α(ν)/ν^2^ of Baltic amber after a slow cooling at a rate of 0.3 K/min down to 12 K. No BP was observed [7]. Different colored lines denote different temperatures.

**Figure 5:**
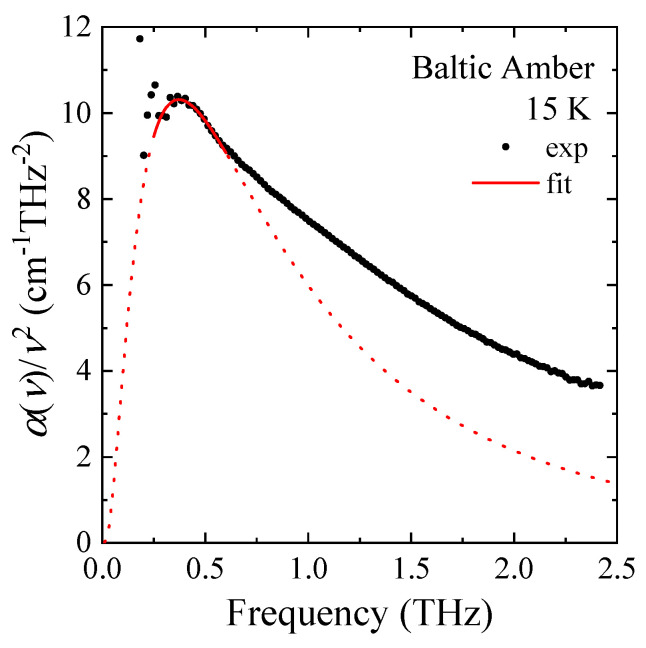
Fitted curve of the BP in THz-TDS spectrum by a log-normal function; α(ν)/ν^2^ of a Baltic amber at 15 K.

In the previous THz-TDS study of a Baltic amber, a BP was not observed upon slow cooling in the standard measurement [7]. The confined water in small pores may cause ice nucleation, and the observation of a BP becomes difficult. In this study, the sample was rapidly cooled down from 300 K to 15 K to keep supercooled water in small pores. After the rapid quench down to 15 K, the sample was heated up to 310 K. Figure 4 shows the temperature dependence of α(ν)/ν^2^, which is proportional to the imaginary part of the complex dielectric constant divided by *n*(ν)·ν of the amber. A clear peak of BP was observed at 0.36 THz and 15 K as shown in Figure 4 and Figure 5. The X-ray inelastic scattering study observed the boson peak at 1.5 meV and 30 K [6]. The energy of 1.5 meV is equivalent to the frequency of 0.36 THz. Therefore, the energy of a boson peak observed by the THz spectrum agrees with that observed by the X-ray in elastic scattering within experimental uncertainty.

In natural amber, the confined water in small pores exists. The melting temperature of the water decreases as the size of the pores becomes small [19,20]. The freezing of confined water occurs below 0 K in a supercooled state. The melting temperature of supercooled water further decreases by rapid cooling. Therefore, the suppression of the ice nucleation is possible by rapid cooling.

**Figure 6:**
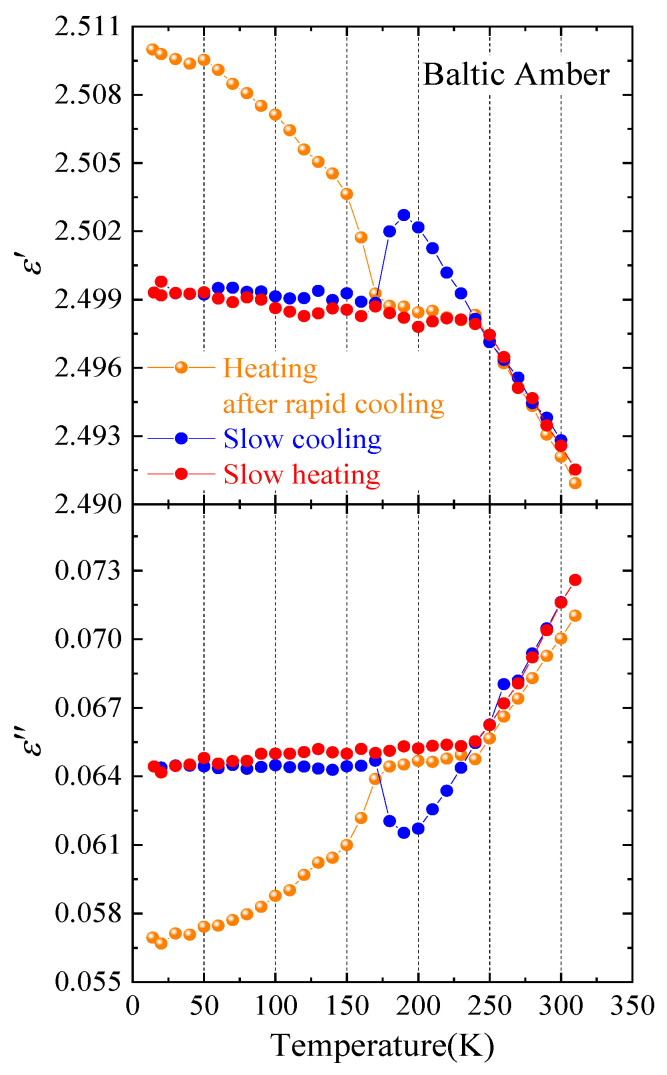
Temperature dependence of real and imaginary parts of the dielectric constant of a Baltic amber at 1.0 THz. Orange, blue, and red circles are heating after the rapid cooling, slow cooling, and heating after slow cooling, respectively.

The difference of real and imaginary parts of a dielectric constant at 1.0 THz between slow and rapid cooling is shown in Figure 6. The thermal hysteresis was observed for slow cooling and subsequent heating processes between 170 and 250 K by the supercooling of the confined water. Slow cooling of the freeze of confined water started at about 250 K in relatively large pores, and the drastic freeze occurred in small pores at about 170 K. Below 170 K after slow cooling, both real and imaginary parts were nearly constant in the frozen ice states. After the rapid cooling at the rate of 5 K/min, the supercooled liquid state reduced down to 15 K, and the subsequent heating real and imaginary parts gradually decreased and increased, respectively. Such changes are caused by the fact that the relaxation time of supercooled water became short upon heating because the dielectric response in the THz range decreases as the relaxation time becomes short. BP is related to the imaginary part of a dielectric constant. The temperature dependence of an imaginary part after rapid cooling showed the minimum at 15 K. This value was much smaller than that of slow cooling as shown in Figure 6. Upon slow cooling at the rate of 0.3 K/min, nucleated small ice may cause an additional response in the THz range and suppressed the appearance of a BP, as shown in Figure 4a. However, the additional response by ice was remarkably reduced in the rapid cooled state, and a BP was observed as shown in Figure 4b.

It was reported that the spectrum of excess low-energy (E∼2–10 meV) density of vibrational states in glasses found by inelastic neutron scattering is described by a log-normal law on the basis of the cluster model of glass structure [21]. The THz-TDS spectrum of α(ν)/ν^2^ of a Baltic amber was fitted by the BP of a following log-normal function [22].

(4) $$I(v)=I_{BP}\times exp(-\frac{{\left(lnv-lnv_{BP}\right)}^{2}}{\sigma^{2}})$$

Here, I_BP_ is a constant. The BP frequency is determined to be 0.36 THz at 15 K as shown in Figure 5. This frequency is comparable with that of a Spanish amber measured by inelastic X-ray scattering [6].

## 4. Medium-Range Order

The crystal shows a translational symmetry by the short-range periodicity related to the lattice constant, which is usually less than a nanometer. In contrast, glass has no short-range periodicity but may exhibit a medium-range order (MRO) on the scale of several nanometers [23,24,25]. MRO is characterized by neutron or X-ray diffraction measurements using the first sharp diffraction peak (FSDP) in the static structure factor S(Q), where Q is the amplitude of a wave vector [5]. It was proposed that the FSDP in the structure factor of network glasses and liquids is a pre-peak in the concentration–concentration structure factor due to the chemical ordering of interstitial voids around cation-centered clusters in the structure [24]. The scale of MRO *L*_m_ is defined by

*L*_m_ = 2π/Q_1_ (5)

and the static structure correlation length *L*_c_ is defined by

*L*c = 2π/ΔQ (6)

where Q_1_ and ΔQ are the peak position and the peak width of the FSDP, respectively. The correlation of a BP frequency with *L*_m_ and *L*_c_ were discussed in various kinds of glasses [5].

Based on the FSDP, two static length scales of Baltic amber have been studied [26]. The scale of MRO, *L*_m_ = 6.0 Å, and the structure correlation length, *L*_c_ = 18.0 Å, were determined at room temperature. *L*_m_, which is the repetitive characteristic distance between structural units, is approximately three times smaller than the value of *L*_c_. The BP correlation length *L*_b_ is defined by

*L*_b_ = *V*_t_/ν_b_, (7)

where *V*_t_ and ν_b_ are the transverse sound velocity and BP frequency, respectively. The temperature dependence of the BP frequency is small and is similar to that of the transverse sound velocity in a glassy state [27]. Therefore, the temperature dependence of the *L*_b_ is also small. *V*_t_ = 1.3 km/s was determined using the values of longitudinal sound velocity *V*_l_ = 2.7 km/s and the Poisson’s ratio σ = 0.36 of Baltic amber at room temperature [28]. *L*_b_ = 35 Å is obtained, and it is about twice *L*_c_.

In an inorganic glass such as 0.22Li_2_O-0.78B_2_O_3_ (LBO22) glass, the FSDP was investigated by neutron diffraction [29]. The *L*_m_ = 4.2 Å and *L*_c_ = 30 Å were determined at room temperature. The boson peak frequency ν_b_ = 1.86 THz and the transverse sound velocity *V*_t_ = 3.4 km/s were determined by Raman scattering and ultrasonic measurements, respectively [30,31]. Consequently *L*_b_ = *V*_t_/ν_b_ = 18 Å was determined. The LBO22 glass has a strong network structure by the boron-oxygen covalent bond and the ionic bond between small lithium ions with strong Coulomb fields and oxygen ions. In contrast, the network structure of amber is weak because the main bonds are van der Walls bonds and hydrogen bonds. The BP frequency and transverse sound velocity of LBO22 is much higher than those of Baltic amber. However, as the common nature in both glasses, the *L*_m_ is much smaller than *L*_b_, and *L*_b_ is approximately half of *L*_c_. It is interesting that such a similarity of characteristic lengths of the intermediate range holds despite the difference in bond strengths of the glass network structures.

## 5. Analysis of Complex Dielectric Constant in the THz Frequency Range

The infrared (IR) spectra from organic polymers reflect the local vibrations of polymer skeletons and attached molecular groups [32]. The difference of IR absorption spectra among Baltic, Kuji, and Dominican ambers between 800 and 4000 cm^−1^ was reported [33]. In the IR region, the mode assignment was studied; for example, the most dominant structures at 2870 and 2930 cm^−1^ were assigned to the symmetric and asymmetric stretch mode of CH_2_ and CH_3_ groups related to van der Walls bonds. The broad absorption band around 3430 cm^−1^ was assigned as an OH-stretch mode related to the hydrogen bond. The Raman scattering study discussed the ν(CC) characteristic of aromatic rings at the 1645 cm^−1^ band [34]. Meanwhile, THz-TDS belongs to the far-IR spectroscopy in the frequency range between 10 and 200 cm^−1^ (0.3~6.0 GHz). The absorption of polymer in a far-IR region is related to heavy groups and/or weak bonds [35]. The terahertz vibrational spectra of disordered systems were analyzed by the fracton, which is a vibrational excitation associated with the self-similar structure of monomers in polymeric glasses [36]. The mode assignment of polymers in a far-IR region is not yet well studied.

**Figure 7:**
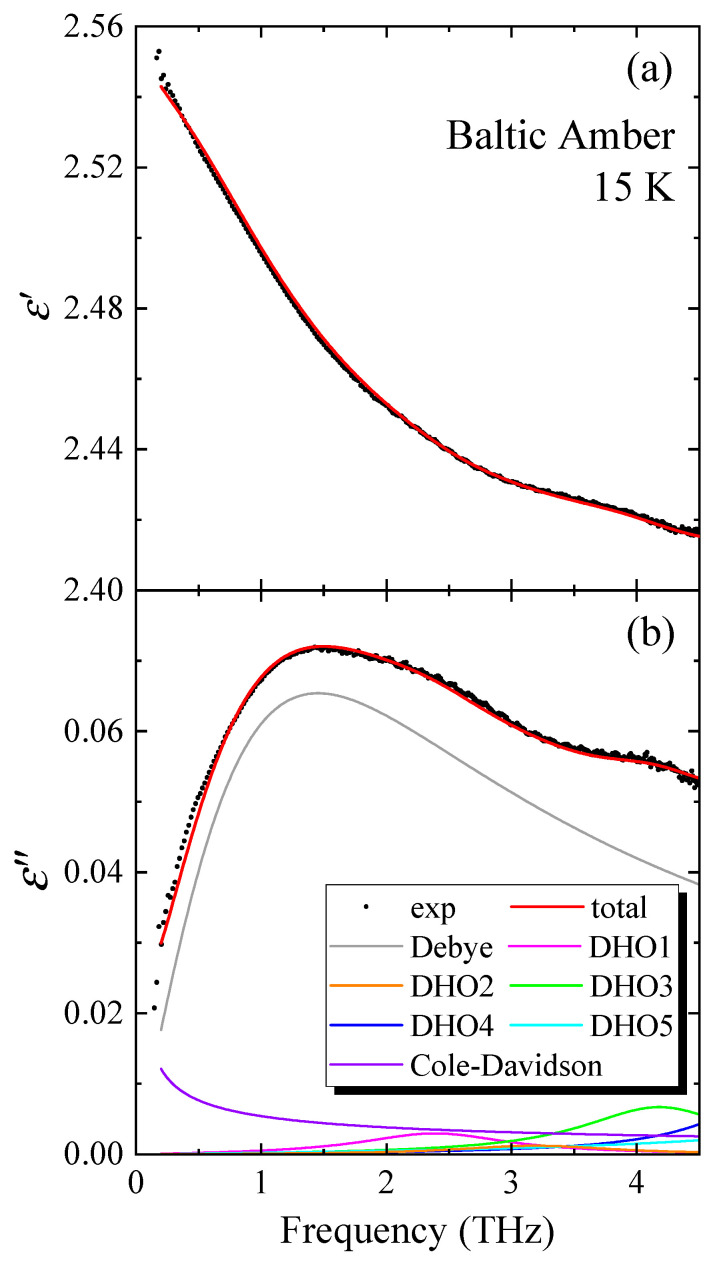
The fitting of (**a**) real and (**b**) imaginary parts of the complex dielectric constant of Baltic amber in the broad THz range using the damped oscillator model (DHO), Debye relaxation, and Cole–Davidson function.

The real and imaginary parts of a complex THz dielectric constant of Baltic amber were reported in the broad frequency range between 0.2 and 6.0 THz [7]. The imaginary part of the dielectric constant, ε″(ν), shows a broad peak at around 1.5 THz and a small peak at around 4.0 THz. Using these broadband spectra, Barden et al. fitted the line shape of all frequency ranges by Debye, Cole–Davidson, and Cole–Cole relaxation functions [37,38]. Such a fitting by a single function may cause the deviation from the observed values, as the frequency increases due to the contribution of various vibrations. Figure 7a,b show the real and imaginary parts of Baltic amber’s complex THz dielectric constant analyzed in the broad frequency range between 0.5 and 4.5 THz using the multiple peak fitting. In this fitting, the damped oscillator model (DHO), Debye relaxation [39], and Cole–Davidson function [40] were used by the following equation.

(8) $$\varepsilon \left(\omega \right)=\varepsilon_{\infty }+\frac{∆\varepsilon }{1-i\omega \tau }+\sum_{j=1}^{5}\frac{S_{j}}{\omega_{0j}^{2}-\omega^{2}-i\gamma_{j}\omega }+A\left(\omega^{0.5}+i\omega^{0.5}\right).$$

Here, $\varepsilon_{\infty },∆\varepsilon ,\;\tau ,\;S_{j},\omega_{0j},\gamma_{j},and\;A$ are the dielectric constant of the high-frequency limit, dielectric constant of the Debye relaxation, oscillator strength of the *j*th mode, mode frequency of the *j*th mode, damping constant of the *j*th mode, and amplitude of the Cole–Davidson formula, respectively. The values of fitting parameters are shown in Table 1.

The observed values and the fitted curve are in good agreement within experimental uncertainty. Because the glass structure of amber is unknown, further investigation on vibrational properties is necessary for the assignment of these multi-peaks.

## 6. Conclusions

Amber is a unique fragile natural glass that has been extensively aged below its glass transition temperature. A Baltic amber that was about 40 million years old was investigated by terahertz time-domain spectroscopy **(THz-TDS)**. It is a typical fragile glass, and the terahertz spectrum of α(ν)/ν^2^ at room temperature showed only the broad wing and no boson peak upon slow cooling. However, after a rapid quench down to 15 K, the intensity of this wing remarkably decreased, and the boson peak was observed at 0.36 THz. The reason for the appearance of a boson peak may be attributed to the suppression of ice nucleation of confined supercooled confined water in small pores. The dynamic correlation length determined by the boson peak frequency in a Baltic amber was compared with the structure correlation length and the scale of MRO determined by the first sharp diffraction peak in the X-ray diffraction (XRD). It was found that the dynamic correlation length determined by THz-TDS was closely related to the static correlation length determined by the XRD analysis.

## Figures and Tables

**Table 1:** Values of the fitting parameters of a dielectric response of a Baltic amber. The fitting equation is Equation (8).

*ε*_∞_	Δ*ε*	*τ*_1_ [ps]	Δ*ε*_2_	*S*_1_ [THz^2^]	*ω*_01_/2*π* [THz]	*γ*_1_ [THz]	*S*_2_ [THz^2^]	*ω*_02_/2*π* [THz]	*γ*_2_ [THz]	*S*_3_ [THz^2^]	*ω*_03_/2*π* [THz]	*γ*_3_ [THz]	*S*_4_ [THz^2^]	*ω*_04_/2*π* [THz]	*γ*_4_ [THz]	*S*_5_ [THz^2^]	*ω*_05_/2*π* [THz]	*γ*_5_ [THz]
2.39	0.13	0.11	0.0054	0.42	2.49	1.47	0.24	3.28	1.55	1.68	4.25	1.52	2.99	5.35	1.31	14.3	8.00	2.52

## Data Availability

The original contributions presented in this study are included in the article. Further inquiries can be directed to the corresponding author.
